# Analysis of the clinical characteristics and predisposing factors for neurological deficit with Hangman fractures

**DOI:** 10.1186/s13018-023-03650-7

**Published:** 2023-03-09

**Authors:** Guangzhou Li, Qing Wang

**Affiliations:** Department of Orthopaedics, The Affiliated Hospital of Southwest Medical University, No. 25 Taiping Street, Luzhou, 646000 Sichuan Province China

**Keywords:** Hangman fractures, Neurological deficit, Clinical feature, Axis, Posterior vertebral wall, Predisposing factor

## Abstract

**Background:**

Hangman fracture is the second most common injury of the upper cervical spine, and neurological deficit with Hangman fracture is not rare. To our knowledge, few reports have statistically analyzed the predisposing factors for this injury. The objective of this study was to describe the clinical characteristics of neurological deficit associated with Hangman fracture and evaluate its risk factors.

**Methods:**

In this retrospective study, 97 patients with Hangman fractures were included. Data on the age, sex, injury etiology, neurological deficits, and associated injuries were obtained and evaluated. The pretreatment parameters, anterior translation and angulation of C2/3, presence of the posterior vertebral wall (PVW) fractures of C2, and presence of spinal cord signal changes were measured. Twenty-three patients with neurological deficits after Hangman fractures comprised group A, and 74 patients without neurological deficit comprised group B. Student’s *t*-test or a nonparametric test and the chi-square test were used to evaluate the differences between groups. Binary logistic regression analysis was used to identify the risk factors for neurological deficit.

**Results:**

Among the 23 patients in group A, 2 were American Spinal Injury Association (ASIA) scale B, 6 were C, and 15 were D, and spinal cord magnetic resonance imaging signal change was observed at the level of C2–C3 disc, C2, or both. Patients with the combination of PVW fractures and ≥ 50% significant translation or angulation of C2/3 were significantly more likely to have a neurological deficit. Both factors remained significant in binary logistic regression analysis.

**Conclusions:**

Neurological deficit after Hangman fractures always presents clinically as a partial neurological impairment. The combination of PVW fractures with ≥ 1.8 mm of translation or ≥ 5.5° of angulation of C2/3 was the predisposing factor for neurological deficit with Hangman fractures.

## Background

Hangman fractures are the second most common injury in the upper cervical spine, accounting for 20–22% of all axis fractures [1–4]. Neurological deficit after Hangman fracture is not rare, and the incidence varies widely [1, 5–8]. However, there are few reports on this injury and little attention given even in recent reports [1, 9–13]. To our knowledge, few reports have focused on the clinical features of neurological deficit after Hangman fracture, the epidemiology, clinical features, pathogenesis, and injury mechanism remain unclear [13], and there has been no statistical analysis published in English of the predisposing factors of this injury. The study’ aim was to describe the clinical characteristics of neurological deficit with Hangman fracture and evaluate its risk factors.

## Materials and methods

### Patients

We reviewed the clinical records of patients with Hangman fractures in our prospectively maintained database between December 2007 and December 2019. Patients were included if they fulfilled all of the following three criteria: (1) Hangman fracture was medically confirmed in the patients; (2) the medical records were complete; (3) lateral plain radiographs and computed tomography (CT) images (including axial-plane CT scans, sagittal- and coronal-plane reconstructions, and three-dimensional reconstructions) of the cervical spine were available. Patients were excluded if they had severe craniocerebral injuries that affected the spinal cord injury evaluation or had malformation, infection, or other cervical spine diseases. This study was approved by the institutional research ethics committee.

One hundred and thirty-three patients who sustained Hangman fractures with or without neurological deficit were reviewed, of whom 36 patients (2 with neurological deficit and 34 without) were excluded, including 31 patients with incomplete medical records or images, 3 with congenital deformity in cervical spine, and 1 with severe craniocerebral injury. Finally, our series included 97 patients; 23 with neurological deficit in the observation group (group A), and 74 patients without neurological deficit in the control group (group B).

Data on the age, sex, injury etiology, fractures types (based on Levine-Edwards classification), neurological deficit, and associated injuries were obtained from clinical records [5]. The neurological deficit severity was assessed by the American Spinal Injury Association (ASIA) scale [14].

### Radiographic assessment

**Fig. 1:**
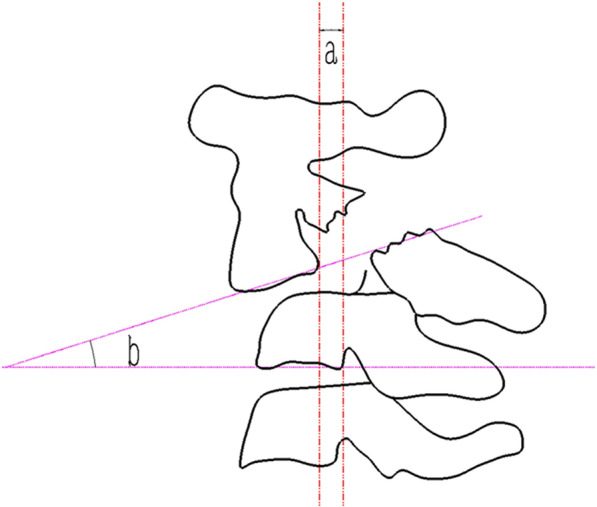
A schematic diagram showing that anterior translation of C2-3 is measured as the distance between lines drawn parallel to the posterior margins of the C3 and C2 bodies at the level of the disc space (**a**), and angulation of C2-3 is measured as the angle formed by lines drawn along the inferior endplate of the C2–C3 vertebrae (**b**)

**Fig. 2:**
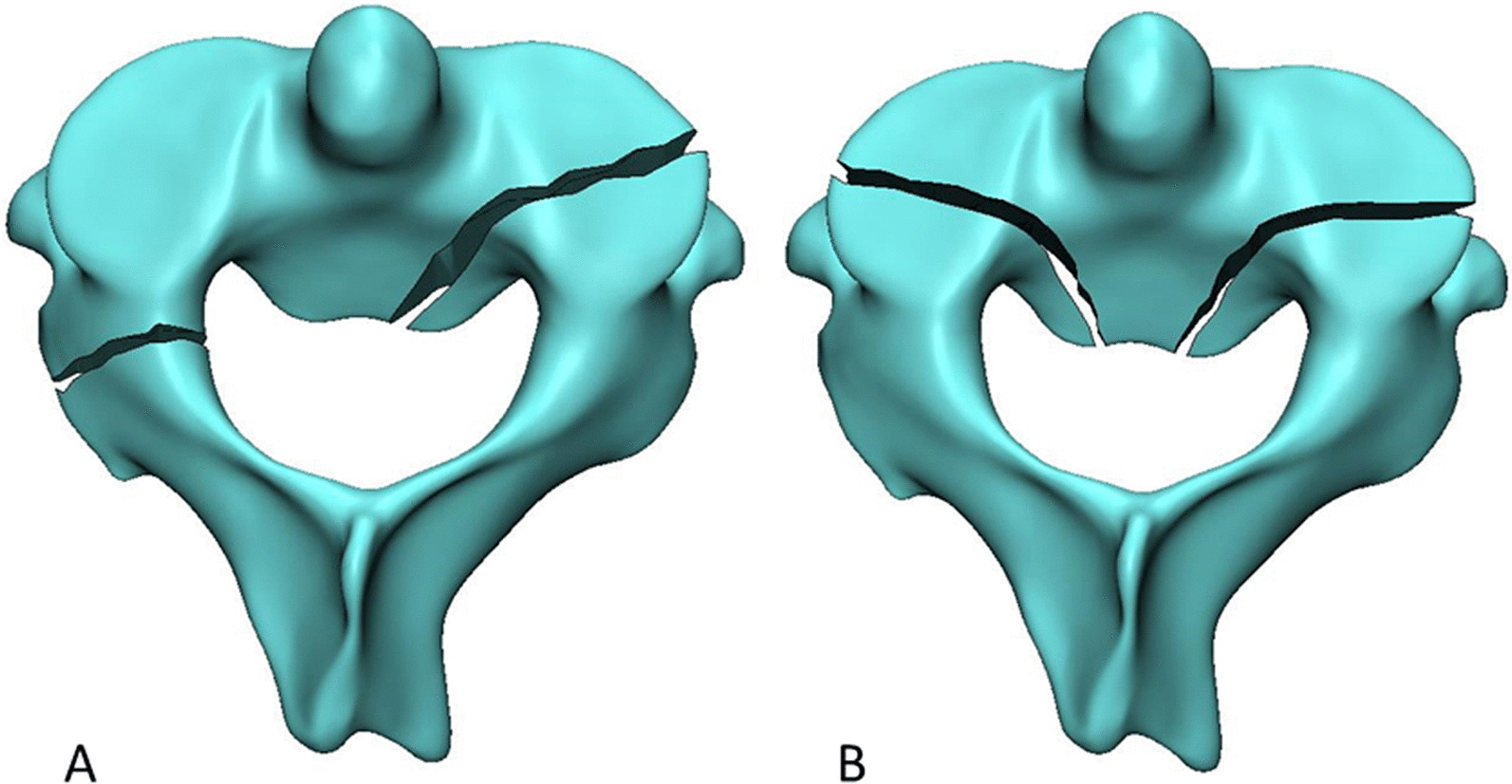
A schematic diagram showing the presence of the posterior vertebral wall (PVW) fracture of C2 on the right side (**A**) or two sides (**B**)

For this review, only pretreatment imaging studies were collected and analyzed. Lateral-view radiographs of the cervical spine were used to measure the C2/3 anterior translation and angulation according to the method described by Li et al. and Watanabe et al. (Fig. 1) [10, 15]. The axial-plane CT scans, sagittal- and coronal-plane reconstructions, and three-dimensional reconstructions were used to look for the posterior vertebral wall (PVW) fracture of C2, which was defined as fracture lines propagating through the posterior wall of the vertebral body of C2 on one or two sides (Fig. 2) [7, 9]. Magnetic resonance imaging (MRI) images acquired in some patients were used to determine if there were spinal cord signal changes, and if so, identify the location and range of the signal changes.

Since a significant anterior translation of C2/3 (≥ 3.5 mm) and/or angulation of C2/3 (≥ 11°) were accepted as radiographic evidence for segmental instability, we divided the translation of C2/3 into 50% (≥ 1.8 mm) and 100% (≥ 3.5 mm) of significant translation to help establish the threshold of parameters for neurological deficit, and we also divided the angulation of C2/3 into 50% (≥ 5.5°) and 100% (≥ 11°) of significant angulation [13]. Then, PVW fractures combined with a different degree of translation of C2/3, as causative factors of neurological deficit, and the presence of PVW fractures and ≥ 1.8 mm and 3.5 mm of C2/3 translation were recorded as PVW fractures combined with 50% and 100% of significant translation, respectively. Similarly, PVW fractures combined with a different degree of C2/3 angulation, as causative factors of neurological deficit, and the presence of PVW fracture and ≥ 5.5° and 11° of C2/3 angulation were recorded as PVW fractures combined with 50% and 100% of significant angulation, respectively.

### Statistical analysis

Statistical analysis was performed in SPSS statistical software (IBM SPSS Statistics for Windows, Version 22.0; IBM Corp., Armonk, NY). Student’s *t*-test or a nonparametric test and the chi-square test were used to evaluate differences between the observation and control groups. Binary logistic regression analysis was used to assess the risk factors for neurological deficit. *P* < 0.05 was considered to be indicative of statistical significance.

## Results

**Fig. 3:**
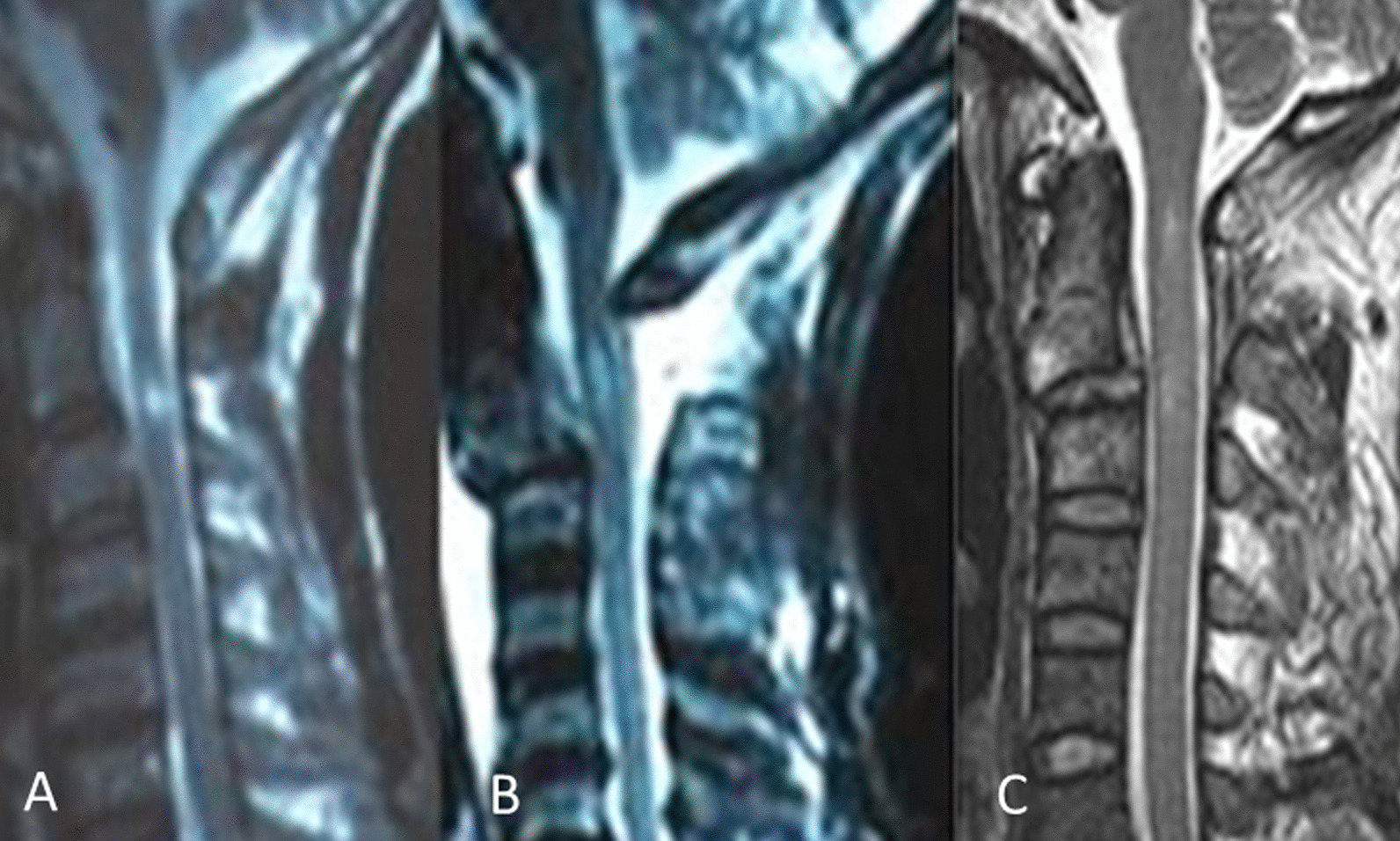
Magnetic resonance images showing the location and range of signal changes on T2-weighted images in the spinal cord. One high signal at the level of C2–C3 disc (**A**). One high signal at the C2 level (**B**). High signals at the C2 and C2–C3 disc levels (**C**)

Among the 23 patients in group A, 2 were ASIA B, 6 were C, and 15 were D [14]. Specifically, 8 of the patients had central spinal cord syndrome, 7 had quadriplegia, 6 had paresthesia, and 2 had monoparesis. Twenty-one patients in group A underwent MRI and 2 did not. High signal intensity on T2-weighted images caused by Hangman fractures was observed in 15 (71.4%) of 21 patients, and there was no abnormal signal in 6 (28.6%) patients. According to the location and range of signal changes in the spinal cord, three types of MRI changes were observed in 15 patients (Fig. 3): one high signal at C2–C3 disc (6 patients) level, one high signal at C2 (5 patients) level, and high signals at the levels of both C2 and C2–C3 disc (2 patients with a continuous abnormal signal, and 2 with non-continuous abnormal signals). Forty-six of 74 patients in group B underwent MRI, and all 46 had no abnormal signal in the spinal cord at level of C2 or C2–C3.

The mean patient ages were 48.4 years in group A (range, 28–89 years; 18 men, 5 women) and 48.2 years in group B (range, 15–82 years; 55 men, 19 women). Age and sex did not differ significantly between the groups (Table 1). In regard to fracture types (based on Levine-Edwards classification), there were 7 type I, 9 type II, 4 type IIa, and 3 type III fractures in group A, and there were 55 type I, 15 type II, 3 type IIa, and 1 type III fractures in group B.

**Table 1:** Clinical data of patients with Hangman fractures

	Group A	Group B	*P* value
Age (years)	48.4 ± 16.5	48.2 ± 14.4	0.969
Sex			0.702
Male	18	55	
Female	5	19	
Etiology			1
High energy trauma	20	63	
Fall, MVA, other	(12, 7, 1)	(36, 23, 4)	
Low energy trauma	3	11	
Fall from standing or a seated height	3	11	
Associated injuries			0.537
Yes	11	30	
No	12	44	
Displacement of C2/3 (mm)	3.9 ± 3.9	1.8 ± 1.7	0.015*
Angulation of C2/3 (°)	9.5 ± 8.1	4.2 ± 5.3	0.006*
PVW fractures			0.283
Yes	22	63	
No	1	11	

*MVA* motor vehicle accident, *PVW fractures* posterior vertebral wall fractures of C2

*Parameter was included in binary logistic regression analysis

When the causes of fracture were divided into high-energy trauma (e.g., fall from a high place, traffic accident, and others) and low-energy trauma (e.g., fall from standing or a seated height), 20 patients (87%, 20/23) in group A and 63 patients (85.1%, 63/74) in group B had experienced high-energy trauma. The proportion of fractures caused by high-energy trauma did not differ significantly between the two groups (Table 1).

Regarding associated injuries, 11 (47.8%) of 23 patients in group A had ≥ 1 associated injuries, including in other parts of the spine (3), cerebral contusions and lacerations (2), clavicle fractures (2), multiple rib fractures (2), extremity fractures (2), and chest injuries (2). Thirty (40.5%) of 74 patients in group B had associated injuries, and the proportion of fractures with complicated injuries did not differ significantly between the groups (Table 1).

The anterior translation and angulation of C2/3 were evaluated in all patients. The mean anterior translation of C2–C3 was 3.9 mm in 23 patients in group A (range, 0–13 mm) and 1.8 mm in 74 patients in group B (range, 0–7 mm), and the anterior translation was significantly higher in group A (*P* < 0.05). The mean angulation of C2–C3 was 9.5° in group A (range, 0°–30°) and significantly higher than the 4.2° in group B (range, − 10°–20°) (Table 1).

In group A, 22 (95.7%) of 23 patients had PVW fractures on one side or both sides of the C2 ring, and in group B, 63 (85.1%) of 74 patients had PVW fractures. The proportion of patients with PVW fractures of C2 was higher in group A, but not significantly (*P* = 0.283; Table 1).

The translation and angulation of C2/3 were included in binary logistic regression analysis, but neither was significant (*P* = 0.101 and 0.077, respectively).

### Combined factors for neurological deficit

When the PVW fracture combined with different degrees of translation of C2/3 was examined as a probable risk factor, 17 (73.9%) of 23 patients and 27 (36.5%) of 74 patients had PVW fractures combined with 50% of significant translation in group A and group B, respectively. The proportion of patients with PVW fractures combined with 50% of significant translation was significantly higher in group A (*P* < 0.05). The proportion of patients with PVW fractures combined with 100% of significant translation was also significantly higher in group A (*P* < 0.05; Table 2).

**Table 2:** PVW fractures combined with translation of C2/3 as causative factors

	Group A	Group B	*P* value
PVW fractures and 50% of significant translation of C2/3			0.002*
Yes	17	27	
No	6	47	
PVW fractures and 100% of significant translation of C2/3			0.003*
Yes	11	13	
No	12	61	

*PVW fractures* posterior vertebral wall (PVW) fractures of C2

*Parameter was included in binary logistic regression analysis, respectively

To help establish the threshold of the translation of C2/3 for neurological deficit, the PVW fracture combined with 50% and 100% of translation of C2/3 were included separately in binary logistic regression analysis. The results showed that a PVW fracture combined with 50% or 100% of translation remained significant (Nagelkerke *r*^2^ = 0.149, *P* = 0.003; Nagelkerke *r*^2^ = 0.118, *P* = 0.005, respectively).

When the PVW fracture combined with different degrees of angulation of C2/3 was considered as another probable risk factor, the proportions of patients with PVW fractures combined with 50% and 100% of significant angulation were significantly higher in group A than in group B, respectively (Table 3).

**Table 3:** PVW fractures combined with angulation of C2/3 as causative factors

	Group A	Group B	*P* value
PVW fractures and 50% of significant angulation of C2/3			0.006*
Yes	13	19	
No	10	55	
PVW fractures and 100% of significant angulation of C2/3			0.001*
Yes	11	13	
No	12	61	

*PVW fractures* posterior vertebral wall (PVW) fractures of C2

*Parameter was included in binary logistic regression analysis, respectively

To help establish the threshold of C2/3 angulation for neurological deficit, the PVW fracture combined with 50% and 100% of angulation of C2/3 were separately included in binary logistic regression analysis. The results showed a PVW fracture combined with 50% or 100% of angulation remained significant (Nagelkerke *r*^2^ = 0.108, *P* = 0.008; Nagelkerke *r*^2^ = 0.172, *P* = 0.001, respectively).

## Discussion

The reported incidence of neurological deficit caused by Hangman fractures has varied and ranged from 6.5 to 25% [9, 11–13, 16, 17], but there have been no reports in English focusing on its clinical features and risk factors. The incidence of neurological deficit caused by Hangman fractures in the current study was 18.8% (25/133), which was consistent with previous studies. In this study, most patients had moderate or mild neurological impairment (15 had ASIA D, 6 had C, and only 2 had B), and the symptoms and signs of these patients varied widely (8 patients had central spinal cord syndrome, 7 had quadriplegia, 6 had paresthesia, and 2 had monoparesis). Considering the high incidence of Hangman fractures in the upper cervical spine injury, clinicians in emergency departments should consider the possibility of Hangman fractures if a patient has these symptoms and signs or complaints about severe pain in the upper cervical spine after trauma [13].

In the present study, MRI revealed for the first time that the signal changes in the spinal cord caused by Hangman fractures could be located at the level of C2–C3 disc (6 patients), C2 (5 patients), or both (4 patients). The MRI data from 9 patients with the signal changes in the spinal cord at the level of C2 showed that fracture of C2 could be associated with neurological deficit.

To assess the risk factors for neurological deficit with Hangman fracture, a series of clinical and radiological parameters were compared between groups. The study results showed that only translation or angulation of C2/3 significantly differed between groups. However, in the binary logistic regression analysis, neither translation nor angulation of C2/3 showed a statistically significant difference. These results revealed that translation or angulation of C2/3 were not predisposing factors of neurological deficit with Hangman fractures.

Previous study reported that PVW fractures was associated with a high incidence of neurological deficit in patients with Hangman fractures [7]. Therefore, PVW fractures combined with different degrees of translation or angulation of C2/3, as causative factors for neurological deficit, were evaluated using the chi-square test between groups in this study. The proportion of patients with PVW fractures combined with ≥ 50% or 100% of significant translation was significantly higher in group A than group B, and the proportion of patients with PVW fractures combined with ≥ 50% or 100% of significant angulation was also significantly higher in group A. Further, in the binary logistic regression analysis, all these combined factors remained significant. The results of this study indicated that PVW fractures combined with 50% of significant translation or angulation of C2/3 could predispose patients with Hangman fracture to neurological deficit.

In the past, bilateral pedicle or par interarticularis fractures of C2 were defined as basic characteristics of Hangman fracture, which caused an enlarged spinal canal. In this situation, only certain severe displacements and angulations of C2/3 (e.g., Levine–Edwards III) could potentially cause neurological deficit, and if a signal change in the spinal cord was observed, the high signal should be located only at the level of C2–C3 disc. However, all of these assumptions caused apparent paradoxes: (1) Levine–Edwards II or IIa or even Levine–Edwards I could also cause neurological deficit, which was often seen in clinical reports or clinical practice [5, 6, 18]; (2) The results of the present study showed that more than half of patients with MRI had signal changes in the spinal cord could located at the level of C2.

The results of this study showed that the prevalence of PVW fractures existed, but only 18.8% of the patients had neurological deficits. Therefore, PVW fracture itself without translation or angulation of C2/3 usually did not cause neurological deficit. The possible explanation for PVW fractures combined with translation or angulation of C2/3 causing neurological deficit is as follows: When fractures occurred, the axis ring was divided into two parts: the C2 body anteriorly, and the arch of C2 surrounding the spinal cord posteriorly; PVW fractures with fracture lines propagating through the posterior wall of the C2 vertebrae on one or two sides constrained the fracture configuration of the C2 ring from expanding instead of causing an enlarged spinal canal; at the moment of injury, an obvious anterior translation and angulation of C2/3 momentarily pulled the spinal cord located in the center of the vertebral arch forward, whereas PVW fractures prevented the spinal cord from moving forward, forming cord impingement and causing neurological deficit [7, 9]. In other words, PVW fracture accompanied with obvious translation or angulation of C2/3 could cause spinal canal narrowing rather than enlarged spinal canal, and spinal stenosis was the cause of neurological deficit.

The statistical results of this study supported our inference, but more importantly, they might establish the threshold of the translation or angulation of C2/3 for neurological deficit: the combination of PVW fractures with ≥ 50% of significant translation (≥ 1.8 mm) or ≥ 50% of significant angulation (≥ 5.5°) of C2/3 could predispose patients with Hangman fractures to neurological deficit. These findings were useful because they could help elucidate the mechanism of neurological deficit with Hangman fractures, and the traditional opinion considering translation and angulation at the C2/3 level and intervertebral disc herniation to be the main causes of neurological deficit might need to be reexamined [5, 6, 19]. Additionally, the instability of C2 itself might need to be considered, especially if PVW fractures are present. It should be mentioned that the maximum displacement and angulation at the C2/3 level occurred at the moment of impact during trauma, and the translation and angulation of C2/3 measured on static images might not be accurate [20]. As in burst thoracolumbar fractures, the maximum canal occlusion and cord compression occurring at the moment of impact and the final static position cannot represent the dynamic process [20].

Some study limitations should be mentioned. First, due to the small sample size, the statistical results might be biased. Second, the instrumentation used for cervical CT and MRI scans was not uniform, which could have led to differences in the assessments of image data.

## Conclusion

Neurological deficit with Hangman fracture always presents clinically as an incomplete neurological impairment. The presence of MRI signal changes in the spinal cord were found in 71% of the patients with Hangman fractures who had neurological deficits, and signal changes were observed at the level of C2–C3 disc, C2, or both. The combination of PVW fractures with ≥ 1.8 mm of translation or ≥ 5.5° of angulation of C2/3 could predispose patients with Hangman fracture to neurological deficits.

## Data Availability

Datasets are available from the corresponding author on reasonable request.

## Abbreviations

PVW: Posterior vertebral wall
CT: Computed tomography
MRI: Magnetic resonance imaging
ASIA: American Spinal Injury Association scale
